# Evidence-Based Practice and Psychological Treatments: The Imperatives of Informed Consent

**DOI:** 10.3389/fpsyg.2016.01170

**Published:** 2016-08-10

**Authors:** Charlotte R. Blease, Scott O. Lilienfeld, John M. Kelley

**Affiliations:** ^1^Centre for Medical Humanities, University of LeedsLeeds, UK; ^2^Program in Placebo Studies, Harvard Medical SchoolBoston, MA, USA; ^3^Psychology, Emory UniversityAtlanta, GA, USA; ^4^Psychology, Endicott CollegeBeverly, MA, USA

**Keywords:** ethics, professional, ethics medical, psychotherapy training, evidence based in clinical psychology, evidence-based practice, informed consent, paternalism

## Introduction

A decade after physicians (including psychiatrists) endorsed the shift toward evidence-based medicine, the world's largest association of psychologists, the American Psychological Association (APA), belatedly but officially embraced the tenets of evidence-based practice (EBP) [American Psychological Association (APA), 2006]. Other clinical psychology associations, including the Canadian Psychological Association, soon followed suit (Canadian Psychological Association, 2012). The interpretation of medical evidence is deeply entwined with ethics; although mainstream medicine has until recently paid relatively little attention to the ethical repercussions of evidence-based practice, the neglect in the field of psychological treatments is even more glaring.

Why does EBP matter for the ethical practice of psychological treatments? Evidence carries ethical imperatives. Both the decision about what is considered to be beneficial in psychotherapy, and the current paucity of research regarding the potential negative effects of psychological treatments, carry ethical implications. We argue that the failure to pay attention to psychotherapy research effectively risks undermining key requisites included in professional codes of practice for clinical psychology, psychiatry, social work, and allied fields. First, EBP bears repercussions for the clinician's duty of professional competence, or what O'Donohue and Henderson (1999) have collectively termed “epistemic duties”—the responsibility to acquire and apply accurate knowledge. Second, EBP is relevant to the duty to respect patient autonomy—namely, the patient's right to make informed decisions concerning his or her treatment plans.

Evidence shows that there are divergent views about the importance, and feasibility, of informed consent among practicing psychotherapists (e.g., Croarkin et al., 2003; Barnett et al., 2007; Goddard et al., 2008). Some of this variation, we argue, probably owes to differences in opinion about what is materially relevant to patients in deciding to undergo psychotherapy; other omissions of informed consent may persist because of continued debate and confusion about what constitutes “evidence” in psychotherapy research and practice. We argue that—despite these challenges—the profession of psychotherapy must find ways to meet the moral obligation of providing adequate informed consent to patients.

## Evidence-based practice and ethical duties

Professional competence—the ability to accurately assess problems, diagnose psychological disorders, recommend an appropriate course of treatment, and successfully carry out that treatment—varies depending on the degree to which the clinician keeps up to date with the latest research and effectively evaluates the evidence. The APA requires that clinicians be trained in EBP to be equipped to appraise the range of evidence regarding the efficacy of different forms of psychotherapy, to recognize the strengths and limitations of clinical intuition, and to understand the importance of patient preferences and values, as well as the relevance of the socio-cultural context in treating clients. In this way, the APA acknowledges that EBP requires knowledge of controlled clinical trials, but also underlines that trial data have inherent limitations. For example, such trials can be unrepresentative of individual patients given that they can be largely insensitive to such factors as age of patient, and comorbidity [American Psychological Association (APA), 2006; cf. Greenhalgh et al., 2014; Sheridan and Julian, 2016]. The APA also emphasizes the importance of keeping up to date with the latest process—and not merely outcome—data on how psychotherapies work [American Psychological Association (APA), 2006].

The duty to be professionally competent carries significant additional implications for the duty to respect patient autonomy. Historically, paternalism was the largely unquestioned bedrock of healthcare practice. Paternalism is defined as “the interference of a state or an individual by another person, against their will, and defended or motivated by the claim that the person interfered with will be better off or protected from harm” (Dworkin, 2010); it was defended on the grounds that doctors were the gatekeepers of medical knowledge, as well as the best judges of how to use that knowledge to serve the interests of patients. Today, healthcare ethics codes (in the West) eschew paternalism: professional clinicians are now obliged to be truthful and to provide adequate disclosure to patients about their diagnosis, the risks and benefits of various treatment options, and their duration and costs (Trachsel et al., 2015; Blease et al., 2016; Trachsel and Gaab, 2016). However, the quality of disclosures to patients depends on practitioner knowledge, illustrating once again why standards of evidence are enmeshed with ethics.

## Evidence of failures in informed consent

Evidence suggests that psychotherapists may be routinely failing to provide adequate informed consent to patients (Dsubanko-Obermayr and Baumann, 1998; Croarkin et al., 2003; Barnett et al., 2007; Goddard et al., 2008). Surveys in the US and UK reveal broad variation among psychotherapists, as well as among psychotherapy schools, in beliefs and practices with respect to information disclosure (Somberg et al., 1993; Croarkin et al., 2003; Martindale et al., 2009). Psychiatrists and adherents of psychodynamic psychotherapy appear to be especially doubtful about the practicability and importance of informed consent (Croarkin et al., 2003; Goddard et al., 2008). Yet even in cases in which therapists routinely disclose information about the specific techniques of therapy—as we later argue—this information may be insufficient for adequate informed consent.

There is evidence that standards of disclosure relate to, and may influence, outcome in psychotherapy. A recent UK study found that patients who reported receiving insufficient information about therapy before it started were significantly more likely to report adverse effects of treatment (Crawford et al., 2016). This finding, although correlational and open to rival interpretations (e.g., therapists who fail to provide informed consent may be less competent in general), supports the notion that the provision of information about therapy helps demystify the treatment process, may reduce anxiety about treatment, and may increase trust between therapist and patient, contributing to better outcomes (Beahrs and Gutheil, 2001; Snyder and Barnett, 2006). It is also conceivable that negative effects may arise from failures to provide understandable information to patients, or that negative effects are a consequence of the manner in which information disclosures are conveyed to patients.

It is worth emphasizing that there are ongoing challenges associated with providing open and honest disclosures in medical practice, and perhaps especially in the context of patients with severe mental health problems, which can sometimes impair judgment, comprehension, or both. At the same time, strong arguments are required to justify paternalistic action in any professional healthcare context. Indeed, even in those circumstances in which health professionals determine that a patient has impaired mental functioning, this does not entail that the duty to provide informed consent be overridden. For example, the UK's Mental Capacity Act of 2005 states that there must be a presumption of capacity for patients to make treatment decisions; in addition, the burden is on health professionals to demonstrate that patients lack any such capacity (UK Department for Constitutional Affairs, 2005). Notwithstanding these pronouncements, when it comes to informed consent there may be practical challenges for psychotherapists who are regularly faced with patients who are extremely anxious, depressed, or agitated as well as those with pronounced psychotic features. The key challenge, then, is to find ways to meet the obligation of adequate disclosure while recognizing the contextual sensitivities involved in providing comprehensible information to patients.

## Explanations for problems with informed consent

Why does informed consent to psychotherapy appear to be “vastly underestimated by many psychologists?” (Barnett et al., 2007). We propose that there are three main reasons for the resistance to informed consent on the part of many practitioners.

### Informed consent is a “process”

First, informed consent to therapy is a process, rather than as a one-time disclosure of information, such as occurs in biomedical contexts. Some psychotherapists may erroneously believe that the procedural nature of understanding how therapy works is a sufficient reason to dismiss or overlook formal disclosure (Barnett et al., 2007). To overcome any such misconceptions, Barnett et al. propose that a combination of written and verbal disclosure of information be provided to patients prior to treatment, but that disclosure should additionally be an ongoing, *active* exchange of information as therapy ensues.

### The complexities of psychotherapy research

Second, psychotherapy research is highly contentious. Compared with the evaluation of psychopharmacological treatments, psychotherapy research is even more difficult to interpret. Debate focuses largely on what constitutes “evidence” in psychotherapy research (Tanenbaum, 2006; Stuart and Lilienfeld, 2007; Goldfried, 2013). Although there is not the space to evaluate and appraise the extensive, ongoing debate about the nature of EBP, we highlight two salient points that we believe transcend this debate, and that are relevant to informed consent to psychotherapy. First, subjective impressions of efficacy based largely or entirely on personal clinical observations can be misleading. A robust body of research strongly suggests that such impressions are frequently inaccurate (Lilienfeld et al., 2014; Casarett, 2016). Second, although there is still disagreement regarding the effectiveness of specific techniques in therapy (e.g., insight-techniques in psychodynamic therapies, or cognitive restructuring techniques in cognitive-behavioral therapy) a large body of research suggests that non-specific factors, such as therapist empathy and the working alliance, should be taken into account when it comes to assessments of psychotherapeutic efficacy. For example, therapist characteristics appear to be important predictors of outcome and in some cases—for example, major depressive disorder—it has been argued that such factors may be more predictive than the specific therapeutic modality (Cuijpers et al., 2008; Wampold and Imel, 2015). Although, this research is controversial, there is widespread consensus among psychotherapy researchers and psychotherapists that—whatever the role of specific factors—the so-called common factors in therapy—are significant mediators of change in treatment (Lambert and Barley, 2002; Huppert et al., 2006; Marcus et al., 2014; Cuijpers, 2016).

### Neglect of research on negative effects

Finally, unlike in pharmacology, evidence of possible negative effects of psychological treatments is both under-researched and largely underappreciated in clinical psychology and allied fields. The routine failure to consider the possible harms of psychotherapy may stem, in part, from intuitive ontological considerations: namely, in psychotherapy the treatment modality involves “talking” rather than the administration of a “physical” treatment such as a drug or surgery (Blease, 2015b). Findings indicate that approximately 10% of patients experience worsening of symptoms following long term treatment in psychotherapy—although it is unclear what proportion of these deterioration effects is due to the treatment, as opposed to a naturally-occurring worsening of symptoms, negative life events outside of therapy, or other influences (Lilienfeld, 2007). In their UK study, Crawford et al. (2016) reported that 1 in 20 patients who enter into psychological therapies report long-lasting negative effects of treatment. At an institutional level, unlike drug treatments in which the FDA requires adverse risks of medications to be investigated and listed, there are no comparable requirements for psychological treatments (Duggan et al., 2014; Markowitz and Milrod, 2015). The longstanding lack of attention to potential harms of psychotherapy may perpetuate the erroneous assumption that psychotherapy carries negligible risk.

## Future directions: what and how to disclose information to patients?

EBP—in its broadest sense—requires therapists to attempt to put aside or find ways to compensate for their biases, and to approach psychotherapy research systematically. Although there is ongoing debate about how to interpret process and outcome research evidence in psychotherapy, there is a duty among therapists not only keep up to date with findings about specific treatments, but to be well-informed about broader debates regarding the potential mechanisms and mediators of therapeutic outcomes. As noted, a wide range of research suggests that explanations for the techniques involved in psychological treatments cannot be taken at face value. For example, given the evidence for the importance of the common factors across different forms of psychotherapy, such as the working alliance, therapist empathy, and the patients' expectations about treatment effectiveness, a strong case can be made for their inclusion in initial information disclosures (Gaab et al., 2015; Blease et al., 2016). It is also likely that there are ways of disclosing the importance of the therapeutic relationship to patients, for example, without undermining that relationship (Blease, 2015a,b; Trachsel and Gaab, 2016), and we strongly encourage research on this issue.

Clients also have a right to be fully informed about the efficacy and effectiveness of specific techniques in therapy. For example, patients with obsessive-compulsive disorder (OCD) have a right to know that exposure and response prevention is the best-supported intervention for their condition—and hence a first-line treatment (Olatunji et al., 2013). Additionally, when it comes to overall efficacy claims, treatment specificity tends to be considerably higher for certain conditions than for others; for example, in contrast to OCD, for which behavioral interventions are the clear treatment of choice, major depression tends to respond to a broad range of psychological treatments (e.g., behavioral, cognitive, interpersonal; see Hollon et al., 2002). Moreover, because certain conditions, major depression again being a prime example, appear to be etiologically heterogeneous, it unlikely that even a highly efficacious intervention will work for virtually all clients. Therefore, clients need to be informed that, depending on their diagnosis, therapeutic interventions may work well for most patients but not all. The point is that research must percolate into disclosure procedures: patients have a right to be furnished with adequate, understandable information about treatment techniques, the importance of common therapeutic factors as well as specific therapeutic techniques, and the risks of harm from a minority of psychological treatments (see Lilienfeld, 2007).

Finally, we recommend that informed consent to psychotherapy is best conceived as a process—initial disclosures of information will require active, ongoing refinement as therapy ensues. Research suggests that including ongoing patient feedback during therapy is one important means of monitoring progress, thereby helping therapists to enhance patient outcomes (Lambert et al., 2001; Sapyta et al., 2005; Shimokawa et al., 2010; Beidas et al., 2015). The bidirectional flow of information about how therapy works, as well as how patients believe therapy is progressing, should be built into the therapeutic process (Barnett et al., 2007).

## Conclusions

Therapists should decisively disavow the pervasive assumption that psychotherapies—although generally effective—carry no risk of harm, and that disclosure (or its omission) somehow carries a different moral valence for psychotherapy than for biomedical treatments. Legally and morally, licensed clinical and counseling psychologists, psychiatrists, and other psychotherapists are duty-bound to eschew healthcare paternalism. Patients deserve to be fully informed if they are to make autonomous choices regarding psychological treatment modalities. Psychotherapy must incorporate best evidence into training and practice if it is to establish and maintain high ethical standards of care. The discussion about how best to accomplish this crucial goal must now begin in earnest.

## Author contributions

CB devised and structured the paper. CB, SL and JK jointly co-authored the content.

### Conflict of interest statement

The authors declare that the research was conducted in the absence of any commercial or financial relationships that could be construed as a potential conflict of interest. The reviewer LF and handling Editor declared their shared affiliation, and the handling Editor states that the process nevertheless met the standards of a fair and objective review.
